# Spotting Signs of Natural Selection

**DOI:** 10.1371/journal.pbio.0020344

**Published:** 2004-09-07

Milk, cheese, and yogurt are so ingrained in the diets of Europeans that it's easy to forget that their ancestors ever ate differently. But about 9,000 years ago, before the domestication of cows, sheep, and goats, milk was a staple only for babies. Back then—just as in most Asian and African cultures today—individuals lost their ability to digest lactose, a sugar found in milk, as they grew up.

But with the domestication of animals, milk became abundant. Among herders, individuals had an advantage if they had versions of genes, also known as alleles, that allowed them to digest lactose into adulthood. They would tend to be healthier and reproduce more than those who could not digest lactose. Thus, by natural selection within herding groups, over generations those who could drink milk into adulthood became more common.

Researchers have found the allele that allows adults to digest lactose, and it's one of the clearest signs of natural selection in humans. In groups with a history of herding, the vast majority of people have the allele, whereas in non-herding groups, most people lack it. Researchers can find such footprints of selection by comparing groups of people that have lived in different environments, for example, or have eaten different diets.[Fig pbio-0020344-g001]

**Figure pbio-0020344-g001:**
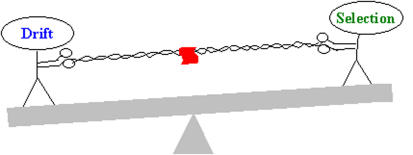


In this issue of *PLoS Biology*, Joshua Akey and colleagues report new-found signs of natural selection in several human genes—including a chunk of Chromosome 7 encompassing four genes, the largest footprint of selection found yet. The research group analyzed the complete sequences of 132 genes in a set of 23 European-Americans and 24 African-Americans. All these genes are involved in inflammation, blood clotting, or blood pressure regulation and were studied as part of a larger project looking for alleles that contribute to disease.

In general, solid evidence of natural selection acting on genes is hard to find. The history of selection can be obscured by a variety of processes. For one, genes can undergo “neutral changes,” in which some of its base pairs change, but without altering the sequence or function of the protein the gene codes for. Also, idiosyncrasies in the history of a population can leave marks on the gene pool. A lineage can go through a “bottleneck,” for example, if a small group splinters off from a larger population and then later multiplies. In general, the splinter group won't perfectly represent the larger population, so the frequencies of alleles for many genes will be skewed in the splinter group's lineage.

Having first ruled out irrelevant changes in genes and population history effects, Akey and colleagues found strong signs of natural selection only in the European-Americans, suggesting this group went through significant changes in climate, diet, or culture more recently than the African-American group. This fits with the well-accepted idea that European populations came from small groups that split off from the larger African population. The researchers find evidence for such an event in the European population about 40,000 years ago. They also estimate that the region of Chromosome 7 was subjected to strong selection around 10,000 years ago, roughly when European herders began drinking milk. Interestingly, two of these genes, *TRPV5* and *TRPV6*, limit the rate of calcium uptake, so selection on one or both of these genes in Europeans could have originated with herding.

Recent studies also found *TRPV6* to be more active in prostate cancer cells. In addition, African-Americans suffer higher rates of prostate cancer, and Akey and colleagues found that European-Americans have alleles of *TRPV6* different from those of African-Americans. Given this evidence, the researchers suggest that this gene may be involved in susceptibility to prostate cancer. This research could therefore shed light on the evolution of complex diseases such as cancer and why different populations suffer different rates of disease.

